# A potential tocopherol acetate loaded palm oil esters-in-water nanoemulsions for nanocosmeceuticals

**DOI:** 10.1186/1477-3155-8-4

**Published:** 2010-02-23

**Authors:** Brian Sheng Xian Teo, Mahiran Basri, Mohd Rezuwan Shah Zakaria, Abu Bakar Salleh, Raja Noor Zaliha Raja Abdul Rahman, Mohd Basyaruddin Abdul Rahman

**Affiliations:** 1 Department of Chemistry, Faculty of Science, Universiti Putra Malaysia, 43400 UPM Serdang, Selangor, Malaysia; 2 Department of Biochemistry, Faculty of Biotechnology and Biomolecular Sciences, Universiti Putra Malaysia, 43400 UPM Serdang, Selangor, Malaysia; 3 Department of Microbiology, Faculty of Biotechnology and Biomolecular Sciences, Universiti Putra Malaysia, 43400 UPM Serdang, Selangor, Malaysia

## Abstract

**Background:**

Cosmeceuticals are cosmetic-pharmaceutical hybrids intended to enhance health and beauty of the skin. Nanocosmeceuticals use nano-sized system for the delivery of active ingredients to the targeted cells for better penetration. In this work, nanoemulsion from palm oil esters was developed as a delivery system to produce nanocosmeceuticals. The stability of the resulting formulation was tested using various methods. In addition, the effect of components i.e. Vitamin E and Pluronic F-68 on the formulation was also studied.

**Results:**

Both vitamin E and Pluronic F-68 were found to co-emulsify and co-stabilized the formulations. The best formulation was found to be the one having the composition of 10% Palm Oil Esters (POEs), 10% vitamin E, 24% Tween 80, 2.4% Pluronic F-68 and 53.6% deionised water. Those compositions are considered to be the best as a nanocosmeceutical product due to the small particle size (94.21 nm), low occurrence of Ostwald ripening and stable at different storing temperatures (5, 25 and 45°C) for four weeks.

**Conclusions:**

Palm oil esters-in-water nanoemulsions loaded with vitamin E was successfully formulated and has the potential for the use as nanocosmeceuticals.

## Introduction

Palm oil esters (POEs) are specialty esters and are emerging oleochemicals in Malaysia as the world's largest exporter and producer of palm oil. In 2008, Malaysia produced 17.73 million tonnes of crude palm oil and 2.13 million tonnes of crude palm kernel oil [1,2]. POEs are wax esters of long chain fatty acids from palm oil esterified with long chain alcohol and have promising revenue as they are highly priced with high profit margins [3]. Besides, they have a wide variety of applications ranging from common uses of wax esters in medicine [4], food [4], lubricant [5] and cosmetics [3,5,6] to some emerging high end products such as, agrochemicals, pharmaceuticals [6] and cosmeceuticals.

The use of POEs in cosmeceutical formulations was introduced in the recent years due to the benefits that POEs can provide, such as excellent moisturizing effect, less greasy and non-irritating [3]. The term cosmeceuticals, coined by Dr. Albert Kligman [7], may be defined as a hybrid of drugs and cosmetics [7,8]. Cosmeceuticals, which are formulated with pharmaceutical-type ingredients [9], have a unique ability to treat or beautify skin from inside out. For the industries dealing in cosmeceuticals, the effectiveness of their cosmetic products is of major concern. The advancement of nanotechnology enables nanoemulsions to be used as a nanocarrier to more effectively deliver the active component in the product, to its targeted cells.

Some of the nanotechnology-based innovations such as nanoemulsions, nano-capsules, nano-pigments and liposomes are widely used in various type of cosmetic products [10]. Solè *et. al*. (2006) define nanoemulsions as emulsion systems having particle sizes ranging from 20 - 500 nm [11]. Due to the small droplet sizes, nanoemulsions are believed to be stable against creaming or sedimentation, flocculation and coalescence [12]. However, Tadros, 2005 also stated that nanoemulsions are vulnerable to instability caused by Ostwald ripening [12].

Vitamin E is the most renowned anti-oxidant known to cosmeceuticals. It is lipid soluble and it helps in protecting membrane lipids from peroxidation when taken orally [7]. Furthermore, it has been shown to decrease sunburn cells after UV exposure, neutralize free radicals, and also act as a humectants when vitamin E is applied on the skin [7]. Although much effort has been concentrated on formulating cosmeceuticals, little has been done to incorporate nanotechnology into cosmeceutical products. The purpose of this research was to formulate stable nanoemulsions system containing vitamin E. This technology utilises nano-sized particles for better penetration as compared to traditional cosmetics [10] which results in the production of nanocosmeceuticals. In addition, the effects of additives i.e. Vitamin E and Pluronic F-68 on the stability of the formulations were also studied.

## Materials and methods

### Materials

Nonionic surfactant, Polysorbat 80 (Tween 80) was purchased from SAFC, U.S.A. The polymeric surfactant, Pluronic F-68 was procured from SIGMA, U.S.A. for simplicity, 10% (w/w) solution of Pluronic F-68 was prepared for the course of this research. The Palm Oil Esters (POEs) were synthesized in our laboratory. The active ingredient, DL-α-Tocopherol Acetate (Vitamin E), is an antioxidant was purchased from FLUKA, Switzerland. Water was deionised by Milli-Q filtration.

### Selective regional study of phase diagram

Palm oil esters (POEs) with a surfactant mixture (mixture of Tween 80 and Pluronic F68 in the ratio of 40:1) were weighed into 10 mL screw-capped glass tube at various weights. Several combinations of ratio of oil and surfactant mixture were made for the study to delineate the boundaries of phases precisely in the phase diagrams. Deionised water was added using the aqueous titration method. The amount of deionised water added was varied to produce the percentage of water in the range of 0% to 100% of total volume at around 5% intervals. The samples were vortexed (Vortex mixer VTX-3000L, LMS, Japan) and then centrifuged at 4000 rpm (1864 g) for 15 mins using a centrifuge (Hermle Z200A, Germany). The samples were then examined visually through cross-polarized light. All the studies were carried out at room temperature, 25°C and 1 atmospheric pressure unless otherwise specified.

### Incorporating DL-α-Tocopherol Acetate (Vitamin E)

Stable emulsion was selected from the homogenous region (1 phase) for the incorporation of DL-α-Tocopherol Acetate. Vitamin E was incorporated into the oil phase by substituting part of Palm Oil Esters. The amount of vitamin E and Palm Oil Esters used were manipulated to ensure that the percentage of oil phase in the emulsion remains constant. This is to ensure that the relative proportions of the water and oil phase were kept constant.

### Nanoemulsions formation

Stable emulsions formed after incorporating of vitamin E was selected for the preparation of 50 g samples. The surfactant mixture was first dissolved into a mixture of POEs and vitamin E via stirring (IKA^® ^- WERKE RW16 basic, Germany) at 150 rpm. Deionised water was added dropwise while stirring at 150 rpm. The system was then homogenized at 250 - 350 rpm for 4 hours. At the end of 4 hours the system was homogenized at 10 000 rpm for 5 mins using high shear homogenizer (PT3100 High Shear Homogenizer, POLYTRON, Kinematica AG, Switzerland).

### Droplet size measurements

The diameter of the droplets for all the formulations was measured using the Particle Size Analyzer (Nanophox, SYMPATEC GmbH, Germany). The size was determined a day after the formulations were formulated. This ensures that the system has achieved equilibrium before the measurement was made.

### Effect of DL-α-Tocopherol Acetate and Pluronic F-68

The effect of DL-α-Tocopherol Acetate and Pluronic F-68 on the nanoemulsions formulations were investigated by manipulating their percentage in the formulations. The effect of these components on the formulations prepared was investigated by carrying out stability test. The stability tests employed in this research are as follows:

### Ostwald ripening

According to Tadros (2005), Ostwald ripening can be quantitatively assessed from the plot of cubic radius of droplet size, r^3 ^versus time, t. Therefore the droplet sizes of all the formulations were measured as a function of time and the slope of the graph plots is the rate of Ostwald ripening. The samples were kept sealed at room temperature.

### Temperature storage

Each formulation was poured into three individual test tubes until three quarters full. Each test tube was kept at a different temperature, i.e. 45°C (placed in an incubator (Shaking Incubator DK-S1020, DAIKI Sciences Co. Ltd, Korea), 25°C (room temperature) and 5°C (refrigerator). The occurrence of phase separations of the system was observed after 24 hours and during weekly observations.

## Results and discussion

### Phase Diagram

**Figure 1:**
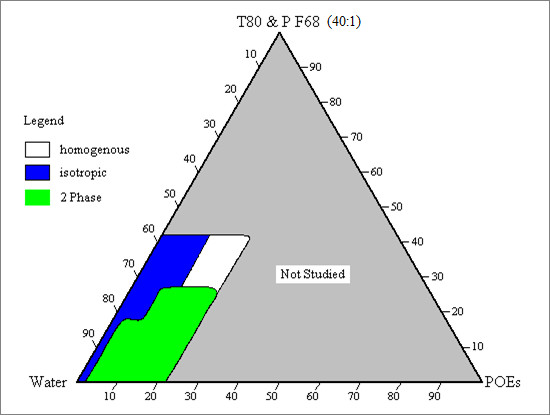
**Phase Diagram of the water/T80: PF68 (40:1)/POEs systems at 25°C**. T80: Tween 80; PF68: Pluronic F-68; POEs: palm oil esters.

The phase diagrams of the water/Tween 80: Pluronic F-68 (40:1)/POEs systems at 25°C, is shown in Figure 1. The phase behavior for this system was determined only for the amount of mixed surfactants and POEs below 40% (w/w) and 20% (w/w), respectively. The oil phase was set not exceeding 20% (w/w) of the whole system so as to reduce the oily texture of the emulsions formed and also to form the desirable oil-in-water (O/W) emulsion. The amount of surfactant was set to be below 40% (w/w) of the whole emulsion because a high percentage of surfactant content was believed to cause irritation to human skin upon contact and also due to the high cost of surfactants.

The surfactant mixture which contained Tween 80 and Pluronic F-68 were mixed at 40:1 weight ratio because thickening and stabilizing properties can be imparted to the emulsion by using just a minute amount of polymeric surfactant. Tween 80 is the primary surfactants in this work as it enables the desired oil-in-water emulsion to be formed. Furthermore, Tween 80 is used in several hundred of pharmaceutical and cosmetic products, owing to its attractive cost and relatively low toxicity [13].

### Incorporating DL-α-Tocopherol Acetate (Vitamin E)

The emulsions system which consisted of 20% (w/w) of POEs, 24.6% (w/w) of surfactant mixture and 55.4% (w/w) of deionised water was chosen from the homogenous region for the incorporation of vitamin E. This emulsion system was chosen because the oil phase was at the maximum of 20% and the surfactant percentage was relatively lower (24.6%, w/w).

The selected emulsion system was modified by adding vitamin E into the oil phase of the system. The percentage of POEs was reduced to 18% (w/w), leaving another 2% (w/w) of the emulsion system for vitamin E. This was to ensure that the relative proportions of the water and oil phase were kept constant, where the percentage of oil phase was 20% (w/w). The centrifugation test showed that this system was stable against centrifugation at 4000 rpm (1864 g) for 15 minutes. Therefore, no further reduction of vitamin E was needed to obtain a stable system.

The selected vitamin E loaded emulsion system consists of 20% (w/w) of oil phase (18% POEs and 2% vitamin E), 24.6% (w/w) of surfactant mixture (24% Tween 80 and 0.6% Pluronic F-68) and 55.4% (w/w) deionised water. Likewise, a stable nanoemulsion containing Ramipril (a potent antihypertensive drug) was successfully formed by Shafiq-un-Nabi and co-workers in 2007 [14]. The nanoemulsion formed consists of 20% oil (loaded with Ramipril), 27% surfactant mixture and 53% water.

### Nanoemulsions formulation

A series of formulations were prepared to study the effect of vitamin E and Pluronic F-68 in the formulations by carrying out the stability test on the nanoemulsions formed.

### Effect of DL-α-Tocopherol Acetate (Vitamin E)

**Figure 2:**
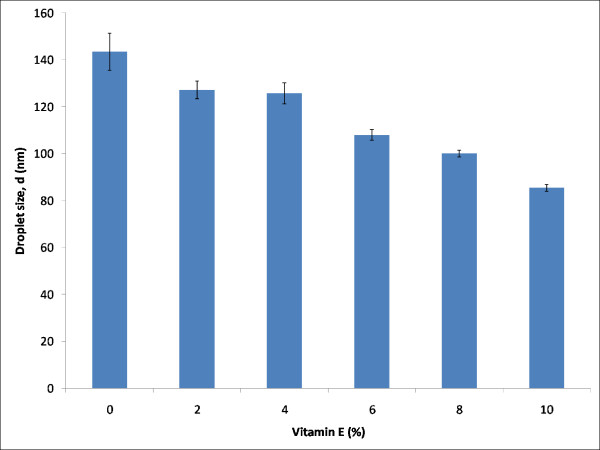
**Effect of increasing percentage of vitamin E on the droplet size at 25°C**.

Table 1 shows the composition of the components in the formulations prepared to study the effect of vitamin E towards the stability of nanoemulsions. Selected vitamin E loaded emulsions system from the previous section were modified by increasing the amount of vitamin E gradually while keeping the weight ratio of the oil phase and the rest of the components in the formulation constant. Figure 2 illustrates the effect of increasing amount of vitamin E on the droplet sizes of the formulations. The mean droplet size for the formulations, F1 to F6 which consisted of 0%, 2%, 4%, 6%, 8% and 10% of vitamin E, respectively, exhibited droplet sizes of 143 nm, 128 nm, 125 nm, 108 nm, 100 nm and 85 nm, respectively.

**Table 1:** Formulations with increasing percentage of vitamin E (0-10%)

Formulation	Composition (% w/w)				
	
	Palm oil esters	Vitamin E	Tween 80	Pluronic F-68	Deionised water
F1	20	0	24	0.6	55.4
F2	18	2	24	0.6	55.4
F3	16	4	24	0.6	55.4
F4	14	6	24	0.6	55.4
F5	12	8	24	0.6	55.4
F6	10	10	24	0.6	55.4

**Figure 3:**
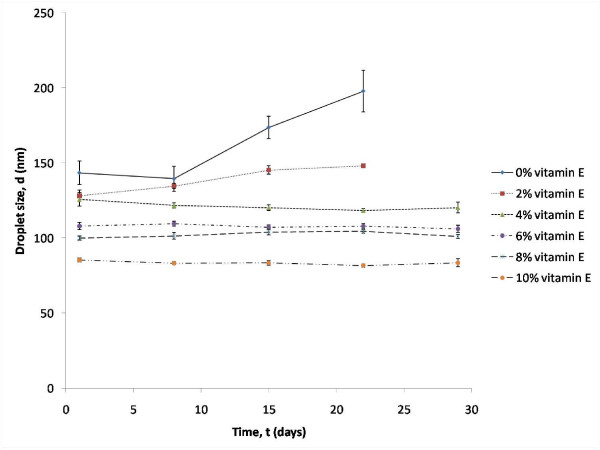
**Effect of time on the droplet size of formulations with increasing percentage of vitamin E at 25°C**.

The results suggested that vitamin E helps to decrease the mean droplet size of the formulations. These observations are in agreement with Pal's research [15] which revealed that droplet sizes decreased when emulsion viscosity was increased. α-tocopherol is a very viscous oil [16] and thus adding more α-tocopherol would increase the viscosity of the emulsions and hence reduced the droplet sizes of the formulation. The ability of vitamin E to stabilize the formulations was also increased as its amount was increased. This property was discovered when the particle sizes of the formulations F1 to F6 were analysed over a period of four weeks. Figure 3 displays the mean droplet size for each of formulations over time (days).

**Figure 4:**
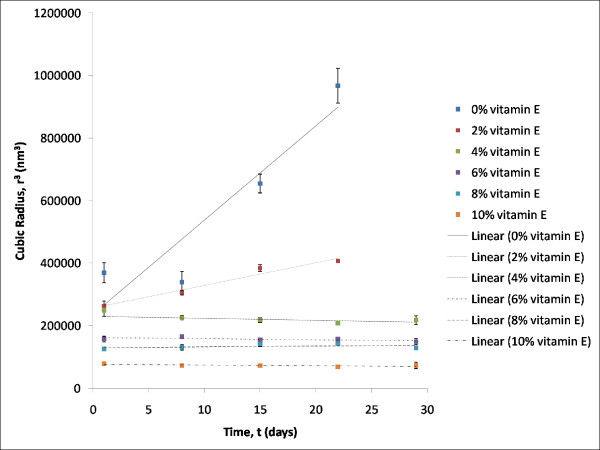
**Effect of time on the cubic radius of formulations with increasing percentage of vitamin E at 25°C: Rate of Ostwald ripening**.

Vitamin E was proven to have stabilizing properties in the formulations which were loaded with 4% (w/w) to 10% (w/w) vitamin E. No significant increment was observed in the mean droplet size when compared to the formulations with 0% (w/w) (F1) and 2% (w/w) (F2) of vitamin E. In addition, formulations F1 and F2 showed phase separation after the third week of analysis. Thereafter, particle sizing analysis was ceased for F1 and F2. The stability of the formulations formed can be confirmed by determining the rate of Ostwald ripening from the graph of r^3 ^against time (day) which is shown in the Figure 4. The rate of Ostwald ripening is indicated by the gradient of the graph plotted. Formulation F1 with 0% w/w vitamin E showed the greatest degree of Ostwald ripening. Experimentally this formulation has the Ostwald ripening rate of 30 137 nm^3^/day, followed by formulation F2 which consists of 2% w/w vitamin E had the Ostwald ripening rate of 7 522 nm^3^/day.

These shows that formulation F1 and F2 were unstable due to the Ostwald ripening process. Rate of Ostwald ripening for the rest of the formulations were not detected due to the negligible changes in their mean droplet size over the period of four weeks. Formulations of F3 to F6 were relatively more stable compared to F1 and F2 within a storage period of four weeks. This had proven that vitamin E has helped to stabilize the formulations and thus it can be regarded as co-emulsifier in these formulations. This is a rather important finding because vitamin E has always been associated as an antioxidant [17] and free radical scavenger [17,18] but has never been extensively reported to have emulsifying properties.

**Figure 5:**
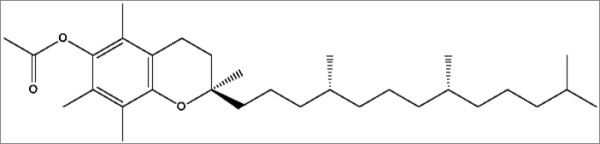
**The molecular structure of DL-α-tocopherol acetate**.

The ability of DL-α-tocopherol acetate (vitamin E) to emulsify and stabilize the emulsions could be due to the presence of the carbonyl and the ether functional groups in the structure of DL-α-tocopherol acetate (Figure 5). The ether group is a hydrophilic group and is capable of forming a hydrogen-bonding network in water through the oxygen atom in the groups [19]. The carbonyl group is believed to behave the same way, thus making the acetate group in the vitamin E structure, a hydrophilic group. Meanwhile, the long chain in the structure of vitamin E is a hydrophobic group and will be dissolved in the oil phase. Consequently, vitamin E is regarded to have a surfactant-like structure, which consists of both hydrophilic and hydrophobic groups. As a result, vitamin E will be adsorbed at the O/W interface and could help to lower the surface tension. Hence, it helps to emulsify POEs in deionised water. Due to this unique property of vitamin E, it can be used in food, cosmetic and pharmaceutical industries especially those in need of a natural emulsifier to substitute current surfactants.

### Effect of Pluronic F-68

**Figure 6:**
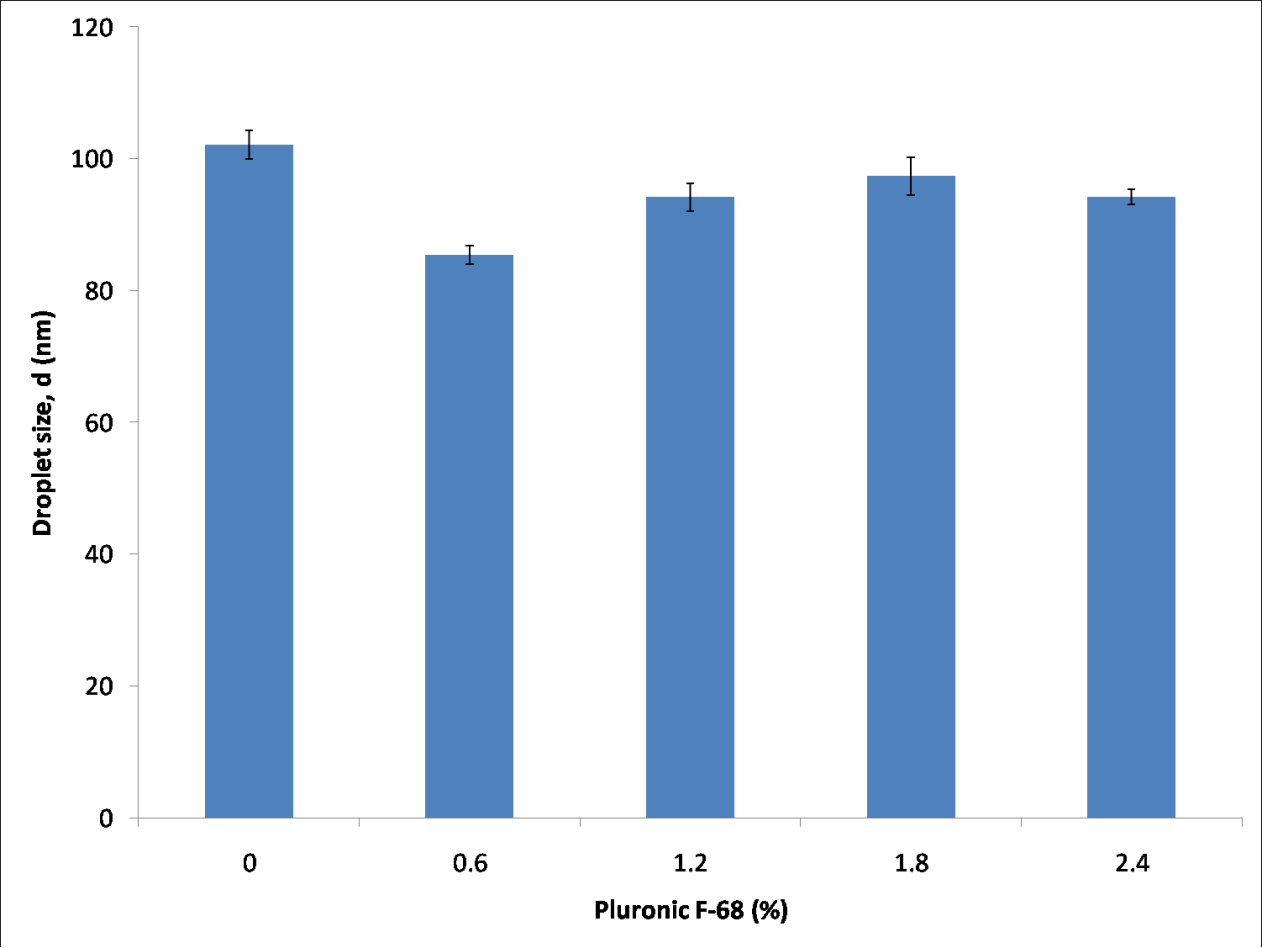
**Effect of increasing percentage of Pluronic F-68 on the droplet size at 25°C**.

Table 2 shows the composition of the components in the formulations prepared to study the effect of Pluronic F-68 towards the stability of nanoemulsions. The percentage of Pluronic F-68 was increased gradually from 0% (w/w) to 2.4% (w/w). Figure 6 shows the mean droplet size of formulations with increasing percentage of Pluronic F-68. Pluronic F-68 exhibited little effect on the droplet size on the formulations when compared to vitamin E. It can be seen in Figure 6 that the formulation without Pluronic F-68 (FP-1) had the mean droplet size of 102 nm, where as with the addition of 0.6% (w/w) of Pluronic F-68 into the formulation (FP-2), decreased the droplet size to 85 nm. For formulations FP-3 to FP-5 which consisted of 1.2%, 1.8% and 2.4% (w/w) of Pluronic F-68, respectively, their mean droplet sizes were approximately 94 nm, 97 nm and 94 nm, respectively. Due to the insignificant difference in droplet size for formulations FP-1 to FP-5, Pluronic F-68 was assumed to have little effect on the droplet size of the formulations.

**Table 2:** Formulations with increasing percentage of Pluronic F68 (0-2.4%)

Formulation	Composition (% w/w)				
	
	Palm oil esters	Vitamin E	Tween 80	Pluronic F-68	Deionised water
FP-1	10	10	24	0.0	56.0
FP-2	10	10	24	0.6	55.4
FP-3	10	10	24	1.2	54.8
FP-4	10	10	24	1.8	54.2
FP-5	10	10	24	2.4	53.6

**Figure 7:**
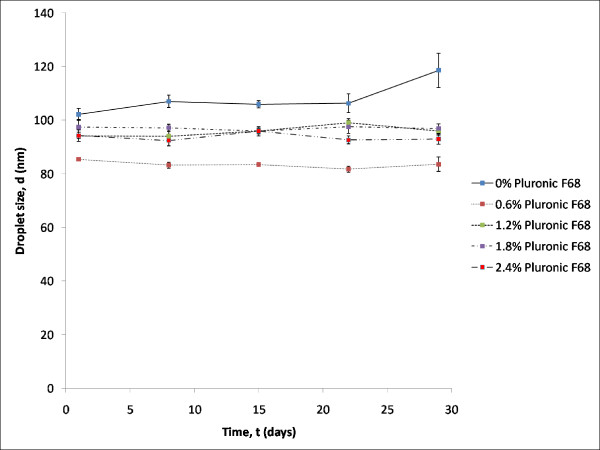
**Effect of time on the droplet size of formulations with increasing percentage of Pluronic F-68 at 25°C**.

However, Pluronic F-68 was discovered to exhibit stabilizing property [20]. This property was shown when the mean droplet size of formulations was analyzed over time. From the graph in Figure 7, it was clearly seen that increasing amount of Pluronic F-68 in formulation FP-2 to FP-5 showed a significant decrease in the growth of droplet size over time as compared to formulations FP-1 (0% (w/w) Pluronic F-68). Formulations with Pluronic F-68 were observed to be stable, as shown by the droplet size of the formulations over time. Pluronic F-68 was shown to be able to stabilize the emulsions. The degree of Ostwald ripening for formulations FP-1 to FP-5 showed the stabilizing ability of Pluronic F-68.

**Figure 8:**
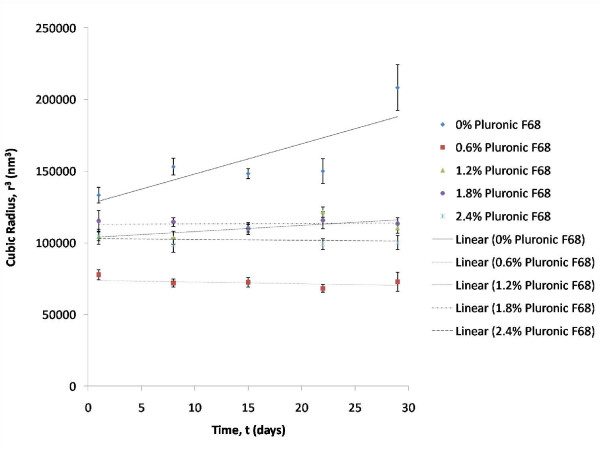
**Effect of time on the cubic radius of formulations with increasing percentage of Pluronic F-68 at 25°C: Rate of Ostwald ripening**.

As shown in Figure 8, the degree of Ostwald ripening for formulations without Pluronic F-68 was indicated with a rate of 2101 nm^3^/day. Rate of Ostwald ripening for the formulation with 1.2% (w/w) of Pluronic F-68 (formulation FP-3) was 428.2 nm^3^/day, whereas the rates of Ostwald ripening for the other formulations were not detected. This proves that Pluronic F-68 was capable of stabilizing the formulations since formulation FP-1 (without Pluronic F-68) had the highest rate of Ostwald ripening. This is consistent with the statement by Tadros (2005) who rationalized that the reduction of Ostwald ripening could be brought about by adding polymeric materials into the emulsions for stabilization purposes [12]. Due to the insignificant effect of the varying amounts of Pluronic F-68 on the stability of the different formulations, it could be concluded that Pluronic F-68 can be used at low concentrations while imparting the same stability.

Pluronic F-68 is a triblock copolymer which serves to stabilize the emulsions through steric repulsion [20]. Furthermore, addition of polymeric surfactants into the emulsions reduces the rate of Ostwald ripening [12]. This is because polymeric surfactants can adsorb strongly at the O/W interface and therefore reduces the surface tension. Addition of Pluronic F-68 into the emulsions has another benefit, that is thickening the emulsions [12].

### Droplet size measurements

**Figure 9:**
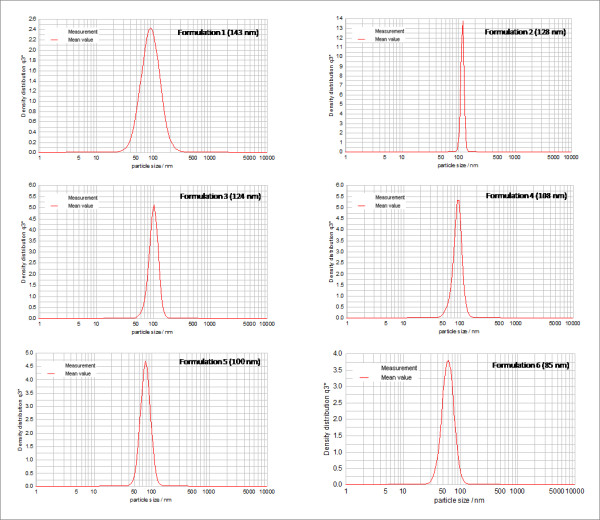
**Particle size distribution graphs for formulations with increasing percentage of vitamin E at 25°C**.

**Figure 10:**
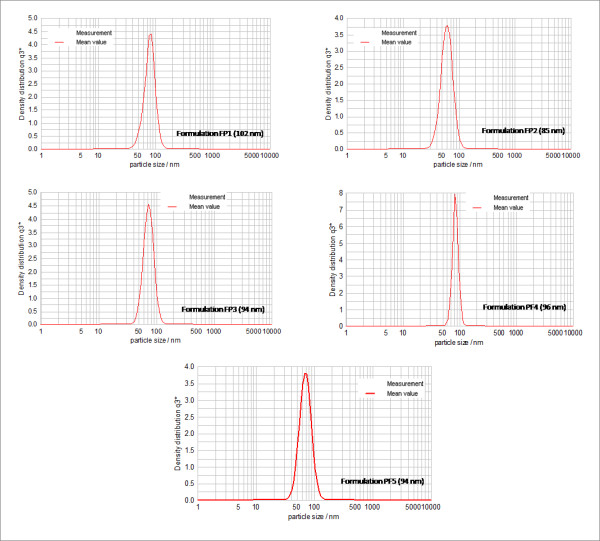
**Particle size distribution graphs for formulations with increasing percentage of Pluronic F68 (0-2.4%) at 25°C**.

Droplet size for each formulation was measured five times at an interval of 7 days between each measurement. All the formulations showed droplet sizes in the range of 80-200 nm, which is within the range of the droplet size of the definition of nanoemulsions by Solè and co-workers [11]. All formulations showed mono-modal peak (mono-dispersed) graphs and most of the peak distributions are narrow suggesting consistent droplet sizes. Figure 9 and 10 illustrates the particle size distribution of each formulation.

### Temperature storage

In this section, it was found that formulation FP-5 was the most stable nanoemulsion system which can sustain temperature up to 45°C within the four weeks of study. Other formulations showed gradual increase in the height of the splitting layers (creaming and sedimentation) when they were stored at 45°C over the duration of 5 weeks. On the other hand, FP-5 also showed no splitting when stored at 5°C and room temperature.

Most of the formulations showed instability when they were stored at room temperature but none of the formulations showed separation at temperature of 5°C within the four weeks of study. This suggests that all the formulations are stable upon storage at 5°C or below. Therefore, all the formulations are suitable to be used as nanocosmeceuticals but they must be stored at low temperatures. However, a more practical nanocosmeceutical product in terms of temperature storage stability can be made from the formulation of FP-5. This is because formulation FP-5 can be used at a wider range of temperature from 5°C to 45°C.

In the storage stability studies, it was found that vitamin E and Pluronic F-68 had improved the stability of the formulations. It was observed in the four-week study that formulation with higher percentages of vitamin E and Pluronic F-68 had lower rates of separation. Thus, vitamin E and Pluronic F-68 have showed the ability to stabilize emulsions against the effect of extreme temperature.

## Conclusions

Stable nanoemulsions containing an appropriate amount of active ingredients were successfully formulated. The best formulation was found to be formulation FP-5 consisting of 10% (w/w) Palm Oil Esters (POEs), 10% (w/w) vitamin E, 24% (w/w) Tween 80, 2.4% (w/w) Pluronic F-68 and 53.6% (w/w) deionised water. It proved to be the most stable in terms of emulsions stability while still containing sufficient amount of active ingredient. Formulation FP-5 is considered to be the most suitable formulation for use as a nanocosmeceutical product because of its particle size of 94 nm and low occurrence of Ostwald ripening. It was found to be stable at temperatures ranging from 5°C to 45°C during the four-week storage stability test. Both vitamin E and Pluronic F-68 were found to co-emulsify and stabilized the formulations.

## Competing interests

The authors declare that they have no competing interests.

## Authors' contributions

BSXT performed all the necessary experiments and analyzed the data collected. MRSZ and MB validated all the experiemental designs and data. MB, ABS, RNZAR and MBAR guided the studies. All authors read and approved the final manuscript.
